# Flux Growth of Single-Crystalline Hollandite-Type Potassium Ferrotitanate Microrods From KCl Flux

**DOI:** 10.3389/fchem.2020.00714

**Published:** 2020-08-20

**Authors:** Fumitaka Hayashi, Kenta Furui, Hiromasa Shiiba, Kunio Yubuta, Tomohito Sudare, Chiaki Terashima, Katsuya Teshima

**Affiliations:** ^1^Department of Materials Chemistry, Faculty of Engineering, Shinshu University, Nagano, Japan; ^2^Research Initiative for Super-Materials, Shinshu University, Nagano, Japan; ^3^Institute for Materials Research, Tohoku University, Sendai, Japan; ^4^Research Center for Space Colony, Tokyo University of Science, Chiba, Japan

**Keywords:** ion exchange, intercalation, hollandite, flux growth, titanate, solid electrolyte

## Abstract

Hollandite-type crystals have unique and interesting physical and chemical properties. Here, we report the flux growth of hollandite-type single-crystalline potassium ferrotitanate (KFTO) with faceted surface features from a KCl flux. We varied the flux growth conditions, including the kind of flux, holding temperature, and solute concentration for growing faceted crystallites. KCl was found to be the best flux to grow the single-crystalline KFTO particles, while heating at or above 900°C was needed to yield the KFTO single crystals. The crystal growth was only weakly dependent on the solute concentration. Next, we characterized the grown single crystals and discussed the manner of their growth from the KCl flux. TEM images with clear electron diffraction spots indicated that the KFTO crystals grew along the <001> direction to form microrods ~10 μm in size. DFT calculation results indicated that the surface energy of the (100) face is lower than that of the (001) face. Based on these characterization results, we proposed a possible growth mechanism of the KFTO crystals.

## Introduction

**Figure 1:**
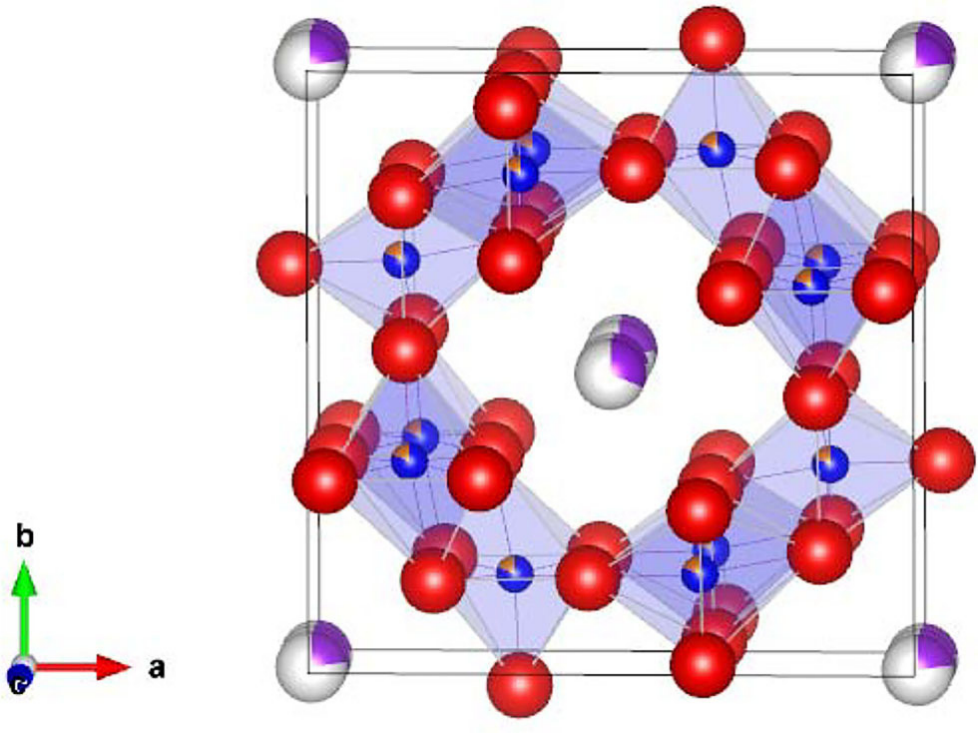
Crystal structure of hollandite-type K_1.75_Fe_1.75_Ti_6.25_O_16_. K, Fe, Ti, and O atoms are represented by purple, orange, blue, and red spheres, respectively. The crystal structure is visualized using the VESTA program (Momma and Izumi, 2011).

Hollandite-type minerals such as silicate exist naturally in the Earth's lower crust and upper mantle and possess significant structural stability (Miura and Iura, 1986). For chemists and physicists, inorganic crystals with hollandite structure are very interesting, because of their unique chemical and physical properties such as fast ion conduction and ion exchange (Michiue and Watanabe, 1999), which allow them to function as solid electrolytes and adsorbents for radioactive elements, respectively (Yoshikado et al., 1995; Aubin-Chevaldonnet et al., 2007; Xu et al., 2016; Tumurugoti et al., 2017; Hassan et al., 2018; Cao et al., 2019). Among these crystals, titanates with hollandite structure possess the stoichiometry of A_x_(B, Ti)_8_O_16_ and tetragonal symmetry, where A is alkali or alkaline-earth cations including K^+^, Rb^+^, and Ba^2+^, and B is a trivalent cation in the oxygen octahedron such as Ga^3+^ and Al^3+^. Here, we focus on the composition K_1.75_Fe_1.75_Ti_6.25_O_16_ (KFTO) from the viewpoints of cost and environmental friendliness. Figure 1 shows its crystal structure. The typical connections of (Fe, Ti)O_6_ octahedra form 1-D tunnel sites along the [001] direction, and the K^+^ cations fill in the center of the empty sites and diffuse into the sites. The walls of such tunnels are formed by (2 × 2) octahedra. Generally, hollandite type materials are synthesized by solid-state reaction (SSR) or hydrothermal reaction (Aubin-Chevaldonnet et al., 2007; Xu et al., 2016; Tumurugoti et al., 2017). However, there are rooms for improvement in crystal quality. Especially, to assess the physical and chemical properties of hollandite type crystals, it is important to prepare KFTO crystals with defined morphology and faceted surface features.

Flux growth is an approach for the nucleation and growth of functional inorganic crystals in molten-salts through the dissolution of ingredients and the precipitation of target crystals. The synthetic flexibility of flux growth conditions can lead to control of size, morphology, and exposed surface features of crystals, and stabilization of intermediate phases (Hayashi et al., 2019). For instance, we have reported the flux growth of platy KTiNbO_5_ (Xiao et al., 2016), cuboidal LiMnO_2_ (Hayashi et al., 2016), and β-Li_2_TiO_3_ (Xiao et al., 2017), which highlights these benefits.

Herein, we comprehensively studied the growth of idiomorphic KFTO crystals from KCl-based fluxes. We explored several flux growth conditions, namely the flux species, holding temperature, and solute concentration in order to yield large KFTO crystals with faceted surface features. The manner of crystal growth in the KCl flux was then studied, and the formation of the single-crystalline KFTO microrods was discussed based on combined experimental and theoretical analysis results.

## Experimental

### Growth of KFTO Crystals

All reagents were purchased from Wako Pure Chemical Industries, Ltd., and used without further purification. K_2_CO_3_, Fe_2_O_3_, and TiO_2_ (anatase, 98.0%) powders were employed as solutes; while KCl, KOH, and KNO_3_ were used as fluxes. In a typical procedure, K_2_CO_3_, Fe_2_O_3_, and TiO_2_ were mixed in the molar ratio of 1.00:1.00:7.14 (giving a nominal composition of K_1.75_Fe_1.75_Ti_6.25_O_16_) and mixed with a flux material at a solute concentration (defined as the molar ratio of KFTO to the flux) between 10 and 80 mol%. The mixture was placed into an alumina crucible with a lid under ambient conditions. Then, the crucible was placed in an electric furnace, heated to a setting temperature of 800–1,000°C at a heating rate of 300°C·h^−1^, and then maintained for 10 h under air. After the holding, the crucible was cooled to room temperature at an arbitrary setting rate under air. The product was washed with deionized water at room temperature and then dried in air at 100°C. The resulting KFTO samples are abbreviated as KFTO_flux_-900, etc. For comparison, KFTO crystals were also prepared via SSR without using the flux according to Equation (1), and the sample is named KFTO_SSR_.

(1) $$\begin{array}{l} 0.875{\text{K}}_{2}{\text{CO}}_{3}+0.875{\text{Fe}}_{2}{\text{O}}_{3}+6.25{\text{TiO}}_{2}\to {\text{K}}_{1.75}{\text{Fe}}_{1.75}{\text{Ti}}_{6.25}{\text{O}}_{16}\\ \;+0.875{\text{CO}}_{2} \end{array}$$

### Characterization

X-ray diffraction (XRD) patterns were recorded on a Miniflex II or SmartLab powder diffractometer (Rigaku) with mono-chromated Cu *K*α radiation (λ = 0.15418 nm). Field-emission scanning electron microscopy (FE-SEM, JEOL, JSM-7600F) images were collected at an acceleration voltage of 15 kV. Nitrogen adsorption isotherms were obtained at −196°C using a Belsorp Mini II sorption analyzer (BEL Japan). For TEM observation, a typical single crystal of KFTO was selected and milled to 5 μm × 5 μm × 100 nm using a focused ion beam apparatus (FIB, JIB-4000, JEOL). The TEM images and selected area electron diffraction (SAED) patterns were taken with the incident beam parallel to the exposed dominate facet at an acceleration voltage of 200 kV. To estimate the chemical compositions of the grown KFTO samples, we carried out elemental analysis. In the elemental analyses, 0.010 g of the KFTOs was dissolved in hydrofluoric acid, and the dissolved solution was analyzed using inductively coupled plasma-optical emission spectrometry (ICP-OES, SII, SPS5510).

The DFT calculations were performed using the generalized gradient approximation (GGA-PBEsol) + U and projector-augmented wave methods, as implemented in the Vienna ab initio simulation package (VASP) (Kresse and Furthmuller, 1996a,b; Perdew et al., 2008). The *U* value for the *d*-orbitals of Fe was set to 4.0 eV (Jain et al., 2011). An energy cut-off of 500 eV and a 2 × 2 × 8 k-point mesh for the bulk were used for the unit cell of 26 atoms in a tetragonal lattice of K_2_Fe_2_Ti_6_O_16_ with *I4/m* symmetry as the starting structure. Relaxation of the crystal structure was allowed in all calculations, and energies of the final optimized geometries were recalculated to correct for changes of the plane-wave basis during relaxation.

To calculate the surface energy of the bulk and surface models, we considered two low-index surfaces [the (100) and (001) facets] and the stoichiometric composition. In the case of (100) facet with Fe-poor termination, we manually arranged the atoms to maintain the stoichiometric composition. The energy of a slab with a surface facet, *E*_slab_, was calculated. The crystallographic symmetry of the top and bottom slab surfaces is essential for achieving a rational computational prediction. The surface energy, γ, is defined as Equation (2).

(2) $$\begin{array}{l} \gamma \;=\frac{1}{2A}\times \left(E_{slab}-N\times E_{bulk}\right) \end{array}$$

where *A* is the surface area of the slab, *E*_bulk_ is the energy per atom in the bulk model, and *N* is the number of atoms in the slab model. The slab cells were constructed from the relaxed bulk structure, and their lattice parameters were fixed. To calculate the surface facets, slab thicknesses >20 Å were chosen for all facets with a vacuum thickness of 20 Å.

## Results and Discussion

### Crystal Growth

**Figure 2:**
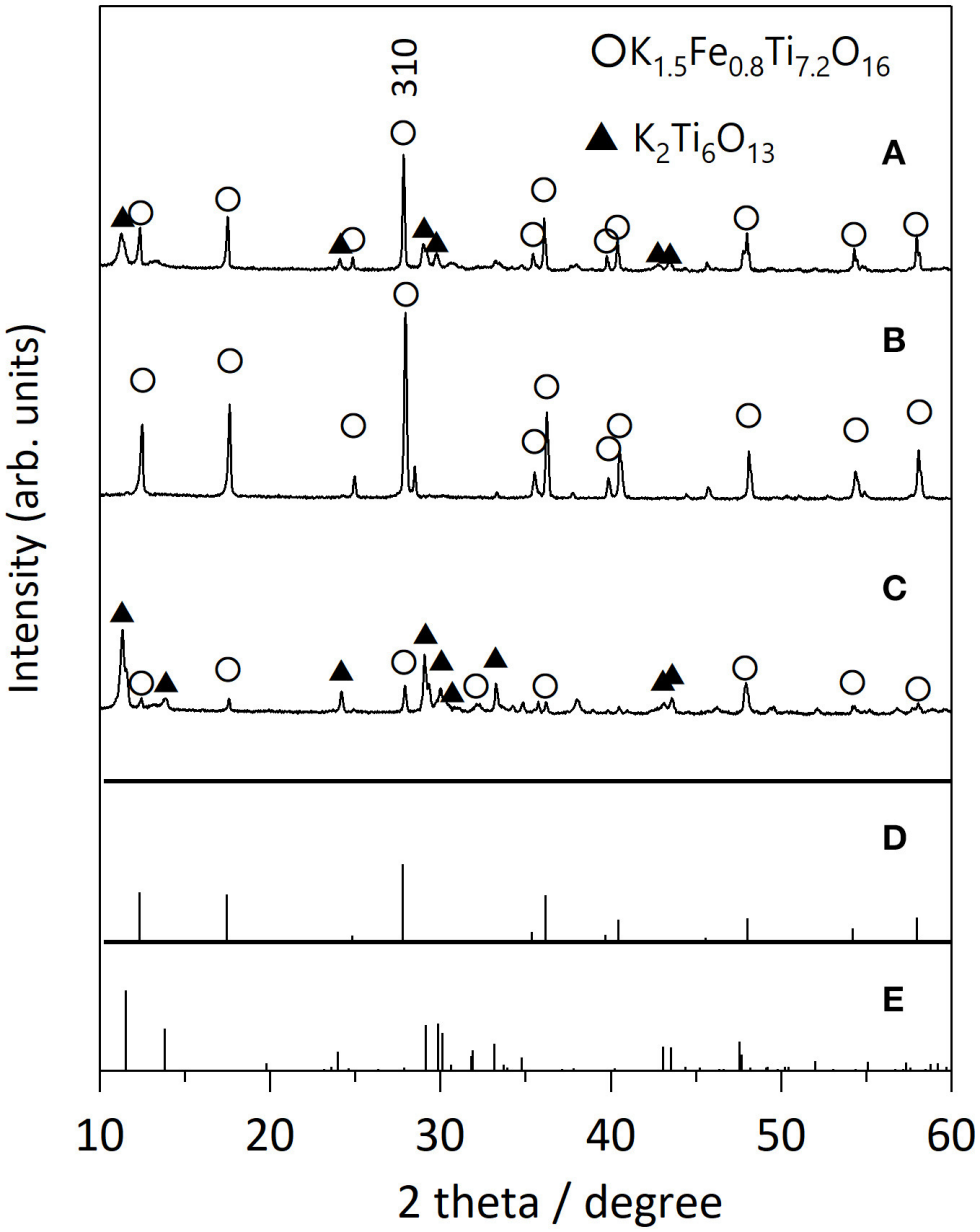
XRD patterns of KFTO crystals grown from various fluxes: **(A)** KCl-KNO_3_, **(B)** KCl, and **(C)** KOH flux. Solute conc., 50 mol%; holding temp., 900°C; holding time, 10 h. The powder diffraction file (PDF) patterns of **(D)** K_1.5_Fe_0.8_Ti_7.2_O_16_ (01-077-0990) and **(E)** K_2_Ti_6_O_13_ (01-074-0275) are from the ICDD-PDF.

We first examined the effect of different flux species on the growth of KFTO crystals, because the cation and anion in the flux can influence the dissolution of the solutes. Figure 2 shows the XRD patterns of KFTO crystals grown at 900°C from KCl-KNO_3_, KCl, and KOH fluxes, whose melting temperatures are 580, 771, and 405°C, respectively. KCl and KNO_3_ were mixed in a 1:1 molar ratio. The respective KCl-KNO_3_, KCl, and KOH fluxes might act as K sources, which is known as self-fluxing. The use of KCl-KNO_3_ and KOH resulted in the formation of K_2_Ti_6_O_13_ as a by-product in addition to KFTO. In contrast, single-phase KFTO crystals were grown from the KCl flux. The difference in product formation could be attributed to the property of the flux, wherein the halide flux would promote the formation of metal complexes in the solution and thereby control the intermediate species. Therefore, from this point on, we only discuss the growth of KFTO crystals from the KCl flux.

**Figure 3:**
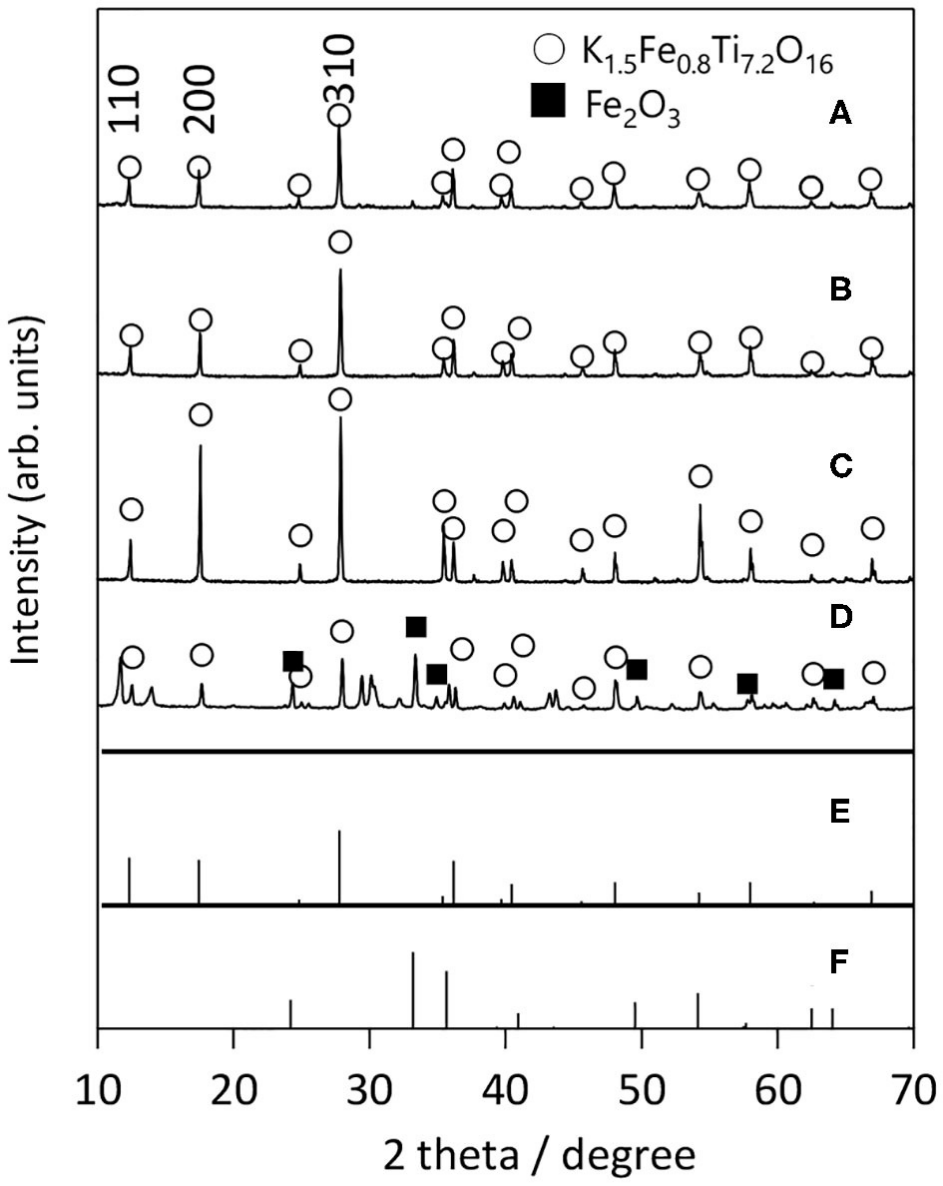
XRD patterns of KFTO crystal grown from a KCl flux at **(A)** 800°C, **(B)** 900°C, **(C)** 1,000°C, and that of KFTO synthesized by SSR at **(D)** 900°C. Solute conc., 80 mol%. The PDF patterns of **(E)** K_1.5_Fe_0.8_Ti_7.2_O_16_ (01-077-0990) and **(F)** Fe_2_O_3_ (01-089-0599) extracted from the ICDD database are also shown.

**Figure 4:**
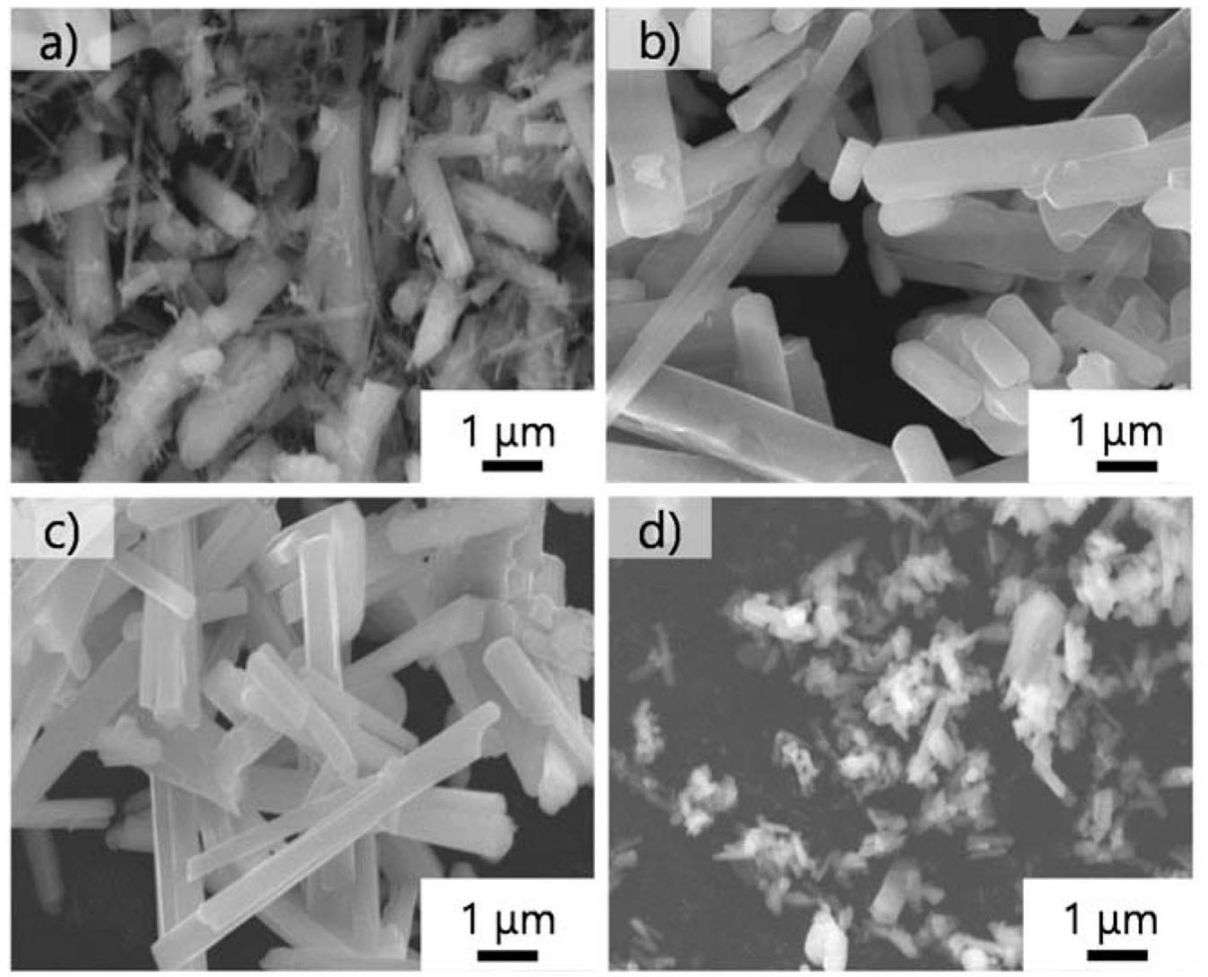
FE-SEM images of KFTO crystals grown at **(a)** 800°C, **(b)** 900°C, and **(c)** 1,000°C, and those solid-state synthesized at **(d)** 900°C. Flux, KCl; solute conc., 80 mol%; holding time, 10 h.

Next, we examined the growth of KFTO crystals under different holding temperatures. Figure 3 shows the XRD patterns of crystals grown from the KCl flux at 800, 900, and 1,000°C (solute concentration: 80 mol%), together with those of KFTO_SSR_ synthesized at 900°C. Heating to 800°C in the presence of flux resulted in KFTO and an unknown phase. Increasing the holding temperature to 900 and 1,000°C eliminated this unknown phase, resulting in the single KFTO phase. On the other hand, SSR at 900°C did not give single-phase KFTO, mainly because the Fe_2_O_3_ did not completely react. Figure 4 is the FE-SEM images of KFTO crystals grown at 800, 900, and 1,000°C and KFTO_SSR_ synthesized at 900°C. At 800°C, both anisotropic undefined crystals several μm in size and needle-shaped crystals were observed. In contrast, heating at 900 and 1,000°C resulted in rectangular crystals with faceted surface features from the KCl flux, and SSR produced small undefined crystals ~100 nm in size. The sharp difference in morphology between the KFTO samples shows two important points. The first is that the present optimum temperature for flux growth (900°C) is ~300°C lower than that for the KFTO prepared by SSR (1,200°C). The second point is that the crystals with well-defined facets were formed possibly due to the formation of intermediate species, as will be discussed later.

**Figure 5:**
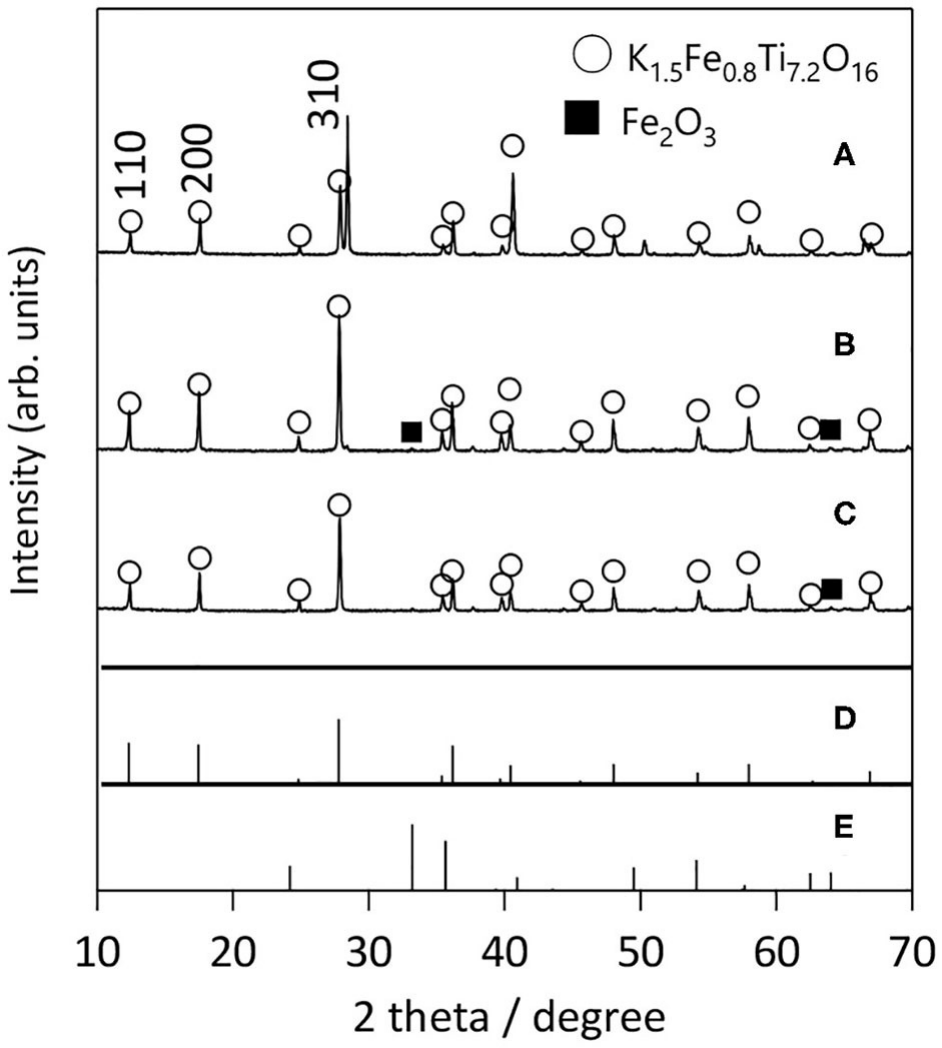
XRD patterns of samples prepared from different solute concentrations: **(A)** 10 mol%, **(B)** 50 mol%, and **(C)** 80 mol%. Flux: KCl; holding temp: 900°C; holding time: 10 h. The PDF patterns of **(D)** K_1.5_Fe_0.8_Ti_7.2_O_16_ (01-077-0990) and **(E)** Fe_2_O_3_ (01-089-0599) extracted from the ICDD database are also shown.

The solute concentration dependence of KFTO crystal growth was next studied, while the holding temperature was fixed at 900°C. Figure 5 shows the XRD patterns of KFTO crystals prepared at 10, 50, and 80 mol% of solute concentrations. At 10 mol%, the diffraction line around 28° could not be indexed to the KFTO phase, showing the formation of the impurity phase. In contrast, at or above 50 mol%, the single-phase KFTO was observed. According to the FE-SEM images (Supplementary Figure 1), the crystal size and morphology were independent of the solute concentrations, as only single-crystalline microrods ~10 μm in size were formed. In summary, we found the best flux growth condition for the KFTO to be: KCl flux, holding temperature of 900°C, and solute concentration of 50–80 mol%. The KFTO samples were named KFTO-80 and KFTO-50 hereafter.

We estimated the chemical compositions of KFTO-80 and KFTO-50. The weight percentages of K, Fe, and Ti for KFTO-50 were found to be 6.2, 13.7, and 41.5 wt%, respectively, while those of K, Fe, and Ti for the KFTO-80 were 6.4, 13.7, and 42.1 wt%. However, the theoretical contents of K, Fe, and Ti for the nominal composition K_1.75_Fe_1.75_Ti_6.25_O_16_ are 9.5, 13.6, and 41.5 wt%, respectively. The error between the Fe and Ti contents of the grown KFTOs and those of K_1.75_Fe_1.75_Ti_6.25_O_16_ is <2%. In contrast, the K contents of the grown KFTO-80 and KFTO-50 were only 66–67% of the theoretical K content of K_1.75_Fe_1.75_Ti_6.25_O_16_. This decreased K content could be attributed to the leaching of K ions during the washing process for the removal of the KCl flux. Formalistically, the chemical compositions of KFTO-80 and KFTO-50 can be expressed as K_1.2_H_0.6_Fe_1.8_Ti_6.2_O_16_ and K_1.2_H_0.5_Fe_1.7_Ti_6.3_O_16_, assuming that the lost K ions were replaced by protons.

### Crystal Growth Process

**Figure 6:**
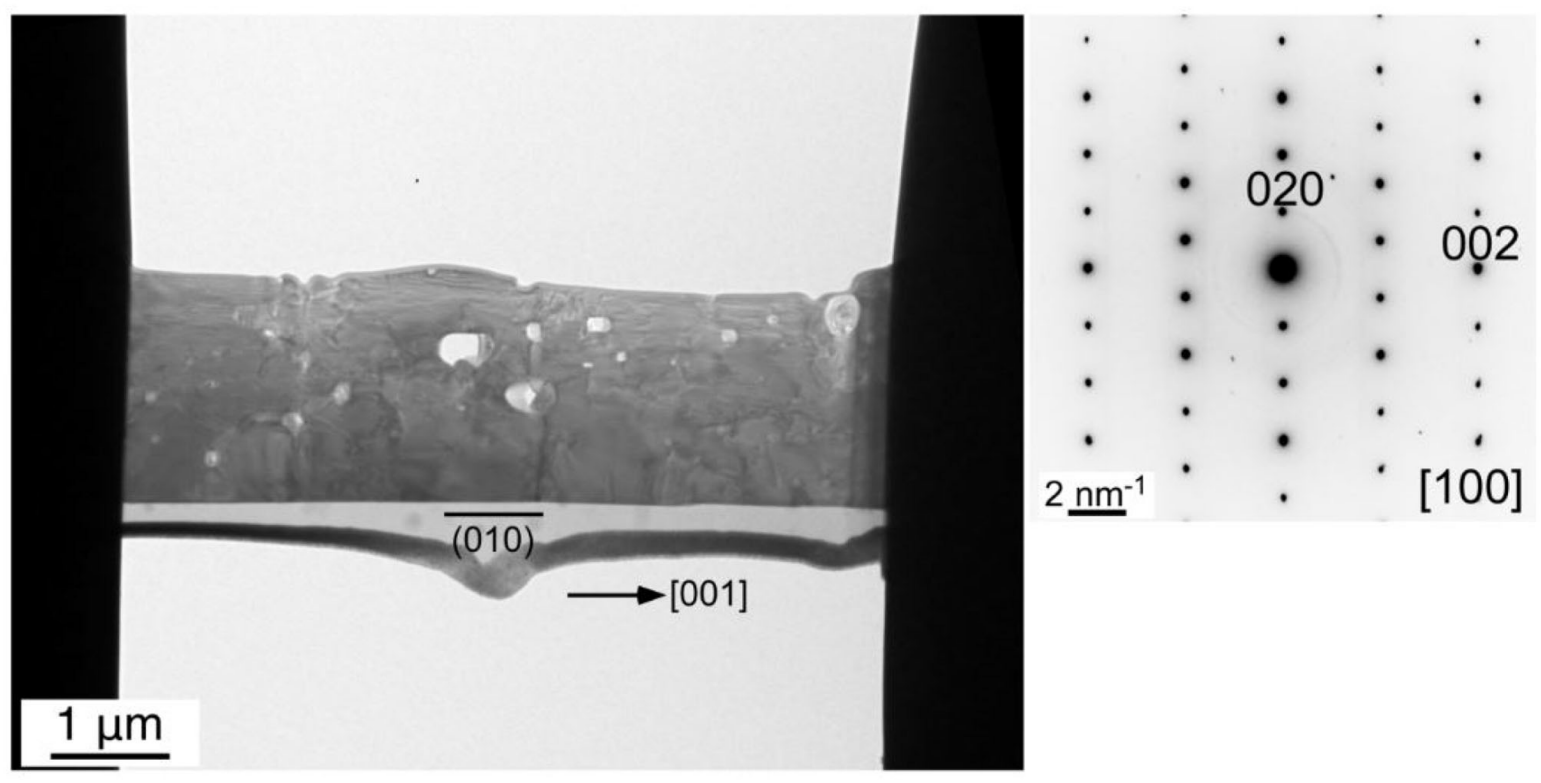
TEM image and the corresponding SAED pattern of the cross-section of a KFTO microrod crystal grown from a KCl flux. Solute concentration: 50 mol%; holding temperature: 900°C; holding time: 10 h.

We carried out TEM observation on the KFTO-50 grown at 50 mol% solute concentration for 10 h at 900°C, in order to determine the crystal growth direction. A typical single-crystalline microrod was processed as described in the Experimental section. The obtained bright-field TEM image and SAED pattern are shown in Figure 6. The ordered diffraction spots taken along the (100) direction indicate that the crystal was single-crystalline in nature. Observation of the exposed facets suggests that the dominant exposed crystal plane at the bottom side was the (010) plane (left image of Figure 6). As a result, the crystal grew along the <001> direction. A similar result was obtained for KFTO-80, as shown in Supplementary Figure 2. Again, the observed clear diffraction spots indicate its single-crystalline nature. Therefore, growth in the <001> direction was found to be dominant regardless of solute concentration.

**Figure 7:**
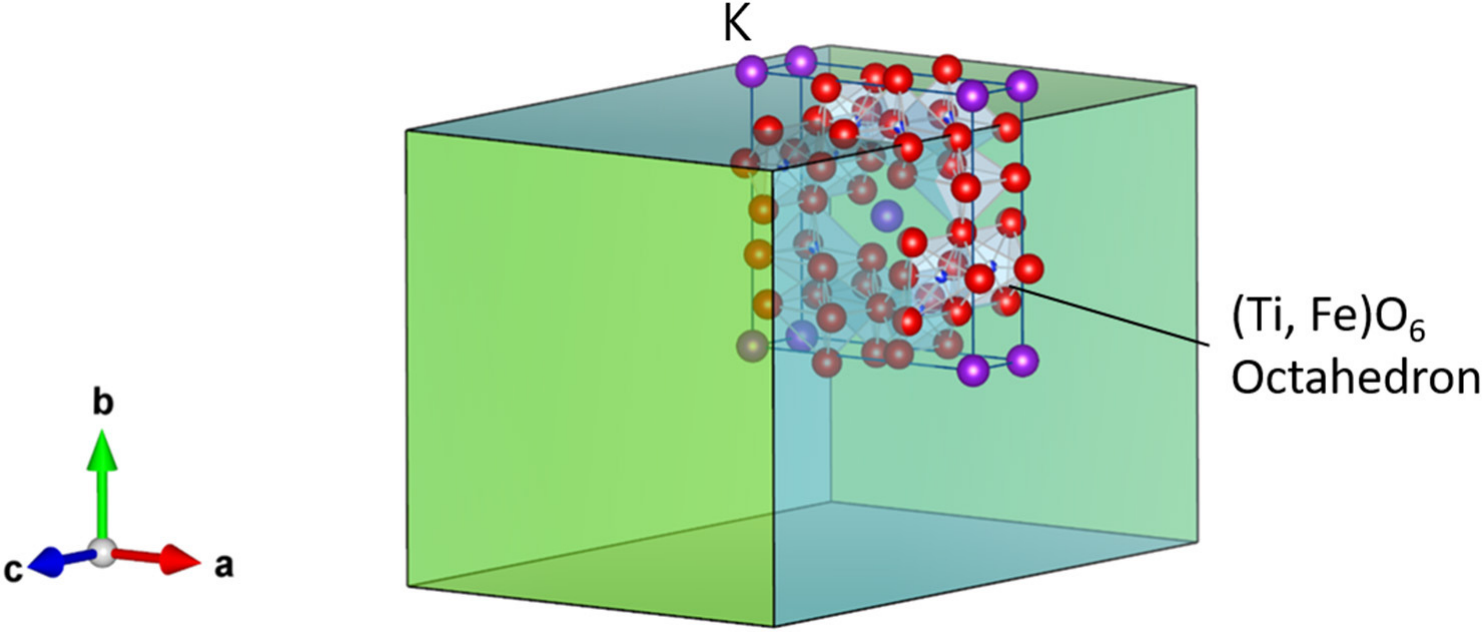
Equilibrium morphology obtained from the Wulff's constructions based on the surface energies derived from the DFT calculation. K, Fe, Ti, and O atoms are represented by purple, orange, blue, and red spheres, respectively.

To explain this preferred crystal growth direction, we calculated the surface energies for the (100) and (001) planes, and the results are shown in Table 1 while the superstructures of KFTO with (100) and (001) facets are shown in Supplementary Figure 3. We found that the surface energy for (100) plane was 0.8414 J·m^−2^, while that for (001) plane was 1.279 J·m^−2^. The higher surface energy of the latter means that the <001> growth direction is reasonable. The calculated surface energies were used in Wulff's constructions to predict the equilibrium morphologies (Wulff, 1901), which are shown in Figure 7. The predicted shape is a rectangular parallelepiped with a longer c-axis. The crystal morphology of the KFTO shown in Figures 4b,c, and Supplementary Figure 1 is a rectangular parallelepiped as well. This similarity in shape indicated that the experimental and theoretical results are highly consistent.

**Table 1:** Surface energy of KFTO for the (100) and (001) facets derived from the DFT calculation.

**Miller index**	**(100)**	**(001)**
Surface energy/J·m^−2^	0.8414	1.279
Surface area for slab model/Å^2^	101.8	60.18

Finally, we briefly propose the possible growth manner of the idiomorphic KFTO microrods. At the initial stage, the KFTO nucleus forms mainly through the solid-state reaction of TiO_2_, Fe_2_O_3_, and K_2_CO_3_. Because Cl^−^ is recognized as a mineralizer in solution-based syntheses through the formation of a metal complex (Bugaris and zur Loye, 2012), the surface of KFTO nucleus undergoes chlorination, followed by the growth of the KFTO crystal along with the <001> direction through the elimination of Cl_2_. The driving force for the flux growth could be the concentration gradient of KFTO resulting from flux evaporation when the mixture was held at 900°C. The present finding opens the door for the growth of hollandite-type crystals using the chloride-based flux.

## Conclusion

The flux growth of KFTO crystals from the KCl-based flux was systematically studied. Among the flux examined, pure KCl was found the most suitable for growing single-crystalline KFTO crystals with surface-faceted features. Holding temperature dependence experiments indicated that the heating at or above 900°C yielded the single-phase KFTO. The crystalline KFTO micro-rods were grown from a KCl, independent of the solute concentration, at 900°C. The TEM observation results indicated the single crystallinity of KFTO and the crystal growth direction along with the <001> direction. DFT calculation result using the slab model of KFTO showed that the surface energy for the (100) facet was lower than that for the (001) facet. The theoretical equilibrium morphology of KFTO in Wulff's construction derived from the DFT calculation was in good agreement with the FE-SEM images of KFTO grown from the KCl at 900°C.

## Data Availability Statement

The datasets generated for this study are available on request to the corresponding author.

## Author Contributions

FH, CT, and KT planned all experiments. KF synthesized the samples. FH, KF, KY, and TS analyzed the single-crystal structures. FH and HS performed the DFT calculation. FH and KT wrote the manuscript and all authors have read and approved it. KT contributed to managing this project.

## Conflict of Interest

The authors declare that the research was conducted in the absence of any commercial or financial relationships that could be construed as a potential conflict of interest.
