# Supplementary material for: Flux Growth of Single-Crystalline Hollandite-Type Potassium Ferrotitanate Microrods From KCl Flux
Source: Front Chem. 2020 Aug 20;8:714. doi: 10.3389/fchem.2020.00714 (PMC7469127; doi:10.3389/fchem.2020.00714)
Supplement: Supplementary file 1 [file Presentation_1.PDF]

Supplementary Material

Flux Growth of Single-crystalline Hollandite-type Potassium Ferrotitanate Microrods from KCl Flux

Fumitaka Hayashi[1], Kenta Furui[1], Hiromasa Shiiba[2], Kunio Yubuta[3], Tomohito Sudare[2], Chiaki Terashima[4], Katsuya Teshima[1,2,4*]

[1]Department of Materials Chemistry, Faculty of Engineering, Shinshu University, 4-17-1 Wakasato, Nagano 380-8553, Japan

[2]Research Initiative for Super-Materials, Shinshu University Nagano, 4-17-1 Wakasato, Nagano 380-8553, Japan

[3]Institute for Materials Research, Tohoku University, Sendai 980-8577, Japan

[4]Research Center for Space Colony, Tokyo University of Science, 2641 Yamazaki, Noda, Chiba 278-8510, Japan

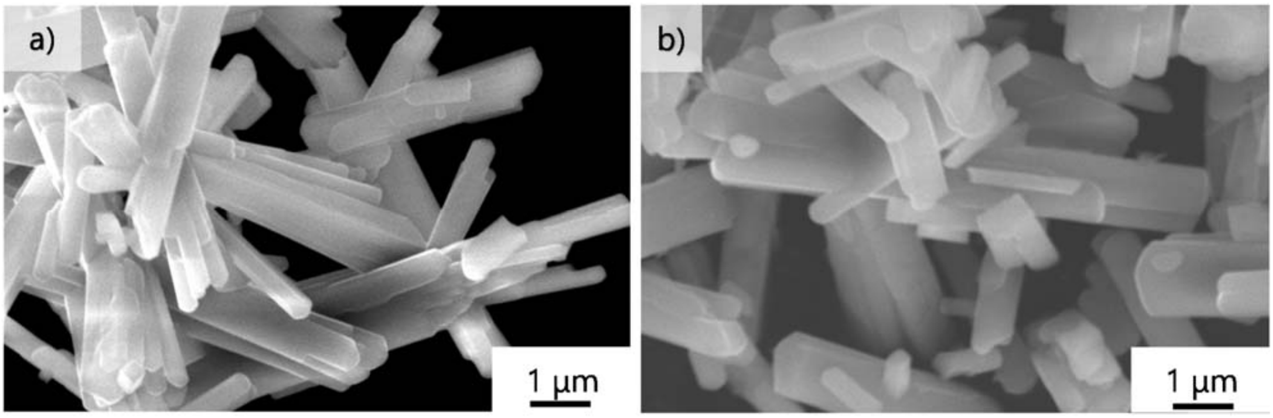

Supplementary Figure 1. SEM images of KFTO crystals grown with different solute concentrations: (a) 10 and (b) 50 mol %. Flux, KCl; holding temp., 900 °C; holding time, 10 h.

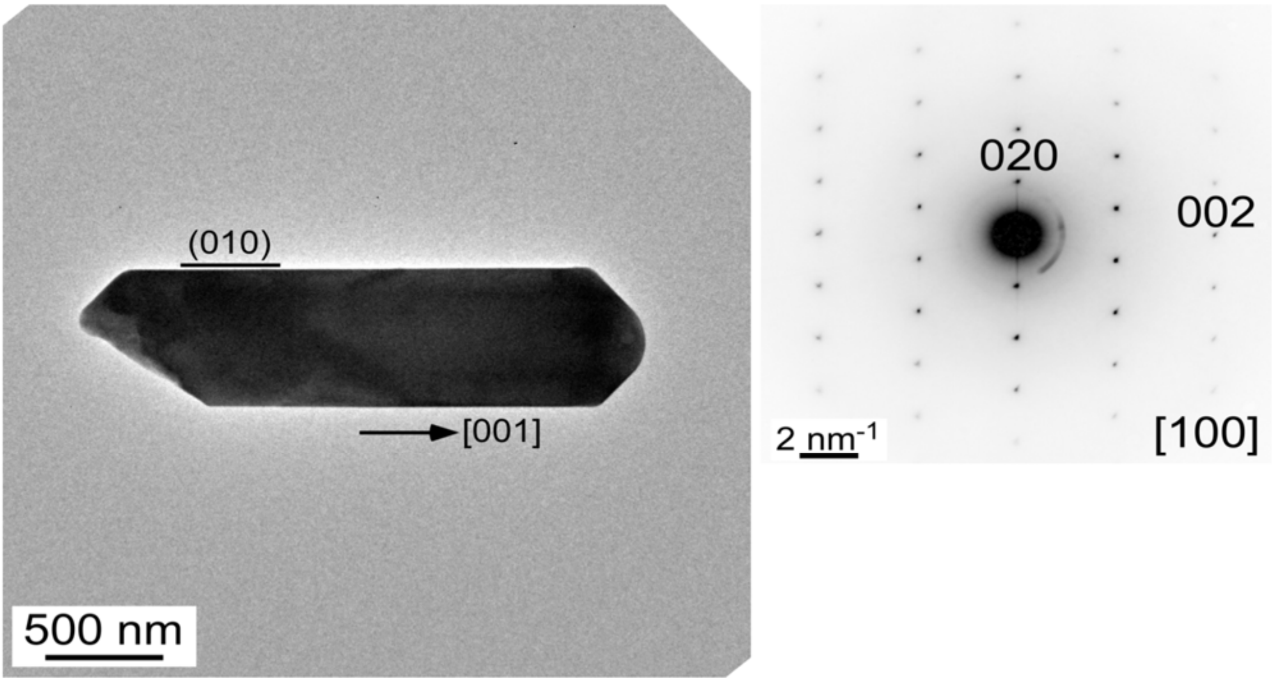

Supplementary Figure 2. TEM image and the corresponding SAED pattern of the cross section of a KFTO microrod crystal grown from a KCl flux. Solute concentration, 80 mol %; holding temperature, 900 °C; holding time, 10 h.

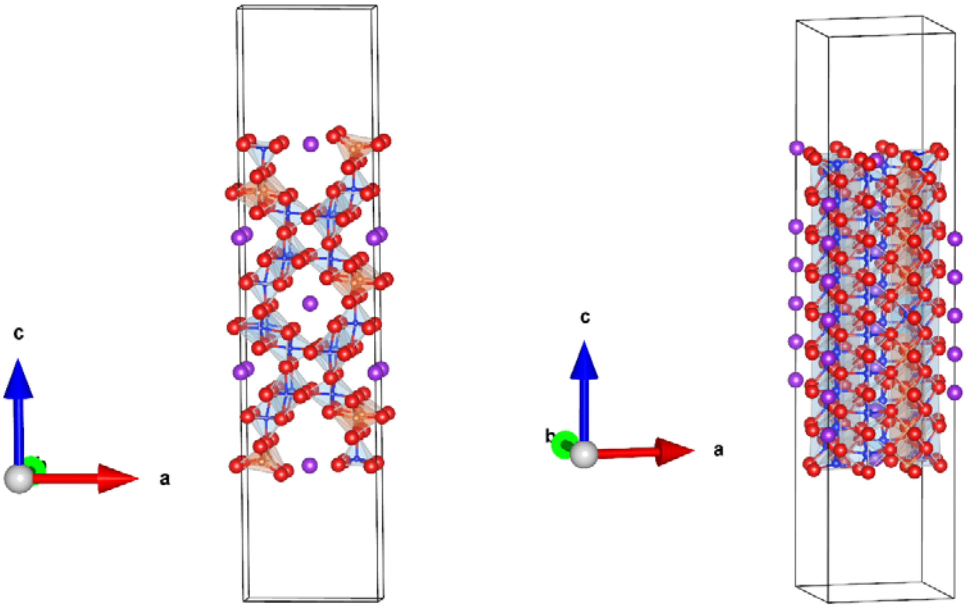

Supplementary Figure 3. Slab models of KFTO showing the (100) (left) and (001) (right) surfaces. Fe, Ti, K, O, and O atoms are represented by purple, orange, blue, and red spheres, respectively.
